# A two-step approach for suprachoroidal delivery of dexamethasone implant using a custom-made injector

**DOI:** 10.1186/s40942-026-00883-6

**Published:** 2026-06-18

**Authors:** Jiyang Tang, Yaoyao Sun, Enzhong Jin, Yuou Yao, Yi Cai, Qiaozhu Zeng, Heng Miao

**Affiliations:** 1 Department of Ophthalmology, Peking University People’s Hospital, Beijing, China; 2 Beijing Key Laboratory of Ocular Disease and Optometry Science, Peking University People’s Hospital, Beijing, China

**Keywords:** Suprachoroidal injection, Dexamethasone implant, Diabetic macular edema, Surgical technique

## Abstract

**Background:**

Suprachoroidal injection enables targeted posterior segment drug delivery with reduced anterior segment exposure. Off-label use of the dexamethasone implant (Ozurdex) via the suprachoroidal route may benefit patients with macular edema who have contraindications to intravitreal therapy. This article aims to introduce a two-step surgical technique for suprachoroidal Ozurdex injection and reports its feasibility and short-term outcomes in an illustrative case.

**Methods:**

A custom‑made injector assembled from a 26‑gauge hypodermic needle and an intravenous catheter. The technique involves: (1) injection of 0.3mL sodium hyaluronate viscoelastic into the suprachoroidal space at 4.0 mm posterior to the limbus; (2) injection of the dexamethasone implant through the same site. The technique was performed in a 37‑year‑old pregnant woman at 24 weeks of gestation with vitreous hemorrhage and macular edema secondary to proliferative diabetic retinopathy in the left eye immediately following pars plana vitrectomy. Main outcomes included feasibility, safety, best‑corrected visual acuity (BCVA), intraocular pressure (IOP) and imaging findings on ultrasound biomicroscopy (UBM) and optical coherence tomography (OCT).

**Results:**

The procedure was completed without intraoperative complications. UBM at postoperative week 1 confirmed Ozurdex within an anechoic suprachoroidal space, which resolved by week 12. BCVA improved from decimal 0.2 (Snellen 4/20) at baseline to 0.4 (20/50) at 12 weeks. IOP remained stable (21–23 mmHg), with no cataract progression or significant IOP elevation. OCT showed resolution of intraretinal cysts.

**Conclusions:**

This two-step technique for suprachoroidal Ozurdex delivery using a custom‑made injector with viscoelastic pre‑injection is feasible and showed no major safety concerns in this case. It may provide an alternative delivery method for selected patients with non‑infectious macular edema who have relative contraindications to intravitreal anti‑vascular endothelial growth factor (VEGF) or corticosteroid injections. Further studies are needed to establish safety and efficacy.

**Supplementary Information:**

The online version contains supplementary material available at https://doi.org/10.1186/s40942-026-00883-6.

## Introduction

Suprachoroidal injection is an emerging treatment option in ophthalmology that allows targeted administration of therapeutic agents to the suprachoroidal space (SCS), achieving higher drug concentration directed at the posterior segment of the eye while minimizing drug exposure to the anterior segment, especially the lens and trabecular meshwork [1]. The current mainstay of therapy for macular edema secondary to non-infectious etiology, intravitreal injection of anti-vascular endothelial growth factor (VEGF) or corticosteroids, is often associated with high treatment burden due to repeated injections and complications such as corticosteroid-associated cataract and glaucoma [2]. For patients with contraindications of intravitreal injections of anti-VEGF therapy or corticosteroids, or for young patients seeking to minimize risk of long-term complication risks, SCS drug delivery route represents an attractive and sometimes the only alternative.

Suprachoroidal injection of triamcinolone acetonide injectable suspension (XIPERE™, Bausch + Lomb, Bridgewater, NJ, and Clearside Biomedical, Alpharetta, GA, USA) 40 mg/mL has been approved by the Food and Drug Administration in the United States for macular edema secondary to uveitis with promising treatment outcomes and relatively lower complication rates [3–5]; however, XIPERE remains unavailable in many parts of the world and the prohibitive cost of XIPERE, including the proprietary microinjector, limits accessibility for patients who might otherwise benefit.

This article describes a novel, simple two-step surgical technique for suprachoroidal delivery of the dexamethasone intravitreal implant (Ozurdex, 0.7 mg, Allergan, USA), a biodegradable solid polymer intravitreal implant that provides controlled dexamethasone release. This technique may be particularly suitable for selected patient population, including pregnant women (in whom intravitreal anti‑VEGF therapy is relatively contraindicated), individuals with a natural lens who wish to minimize cataract progression risk, patients with a history of steroid‑induced intraocular pressure (IOP) elevation, and those with chronic conditions requiring sustained steroid release. To illustrate the feasibility and short‑term safety of this technique, we present a representative case of a pregnant patient with diabetic macular edema (DME) who underwent the procedure immediately following pars plana vitrectomy.

## Methods

The surgical technique described in this report was performed on a 37-year-old female at 24 weeks of gestation with vitreous hemorrhage and macular edema secondary to proliferative diabetic retinopathy in her left eye. The study was conducted in accordance with the Declaration of Helsinki for research involving human subjects. This study and the off-label use of Ozurdex were approved by the Ethic Committee of Peking University People’s Hospital. Informed consent has been obtained from the patient. The patient underwent best corrected visual acuity (BCVA), Goldmann applanation tonometry, slit lamp microscopy, indirect ophthalmoscopy, fundus color photography and optical coherence tomography (OCT) examinations before surgery; on post-operative 1-week and 12-week follow-up visits the patient received the aforementioned examinations in addition to ultrasound biomicroscopy (UBM).

BCVA was examined using a standard logarithmic visual acuity chart; OCT radial scans centering on the fovea were performed with M-400 K BMizar OCT (TowardPi Medical Technology, Beijing, China). Fundus color photography and fluorescein angiography were performed using CLARUS 700 (Zeiss Meditec, Inc., Dublin, California, USA); ultrasound biomicroscopy (UBM) was performed with Aviso (Quantel Medical, Inc., Bozeman, MT, USA).

### Fabrication of custom-made SCS injector

**Fig. 1:**
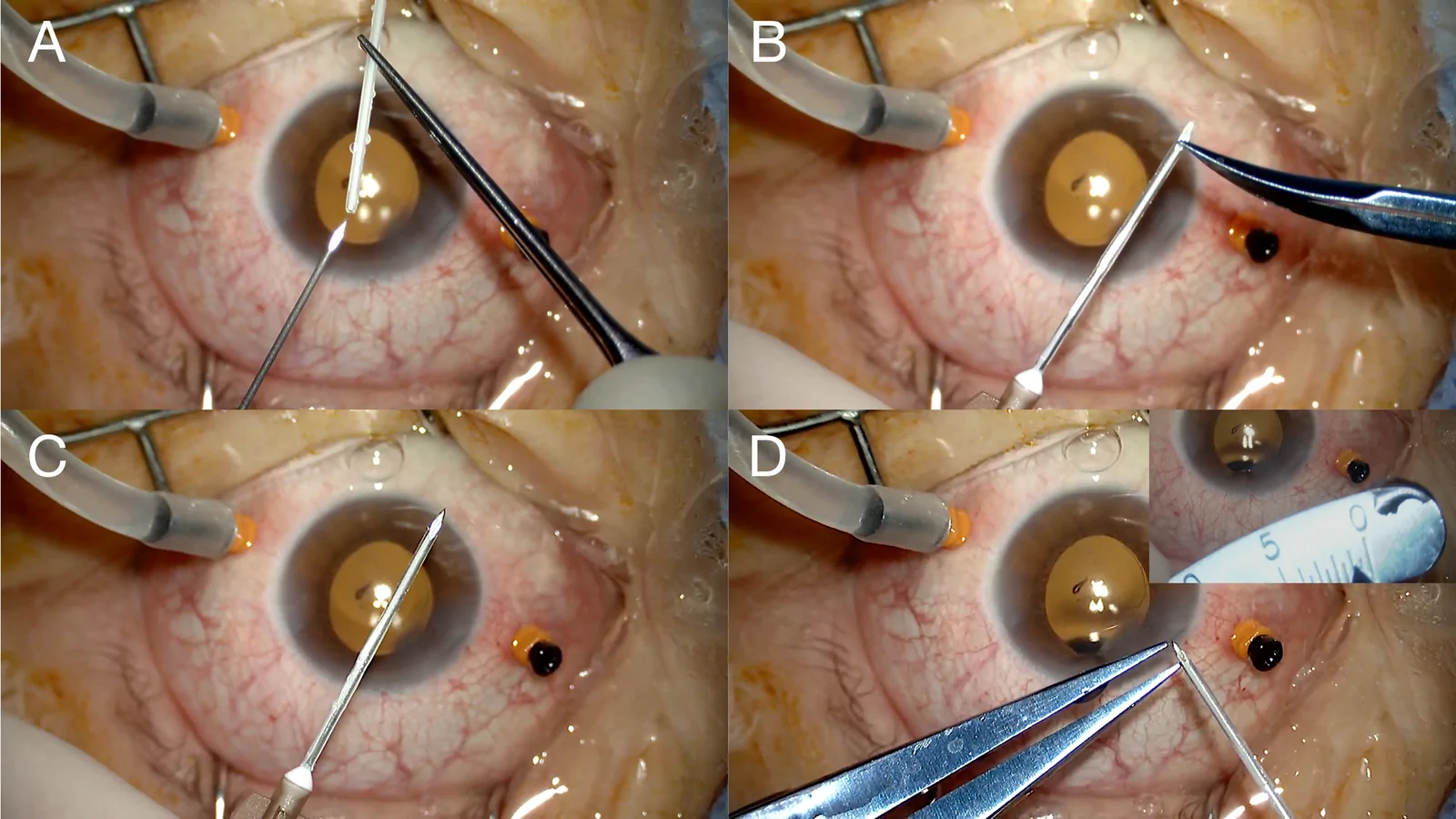
Fabrication of custom-made SCS injector. **A**, the 26G intravenous catheter for single use was sheathed onto the 1mL sterile hypodermic syringe. **B**, ophthalmic micro-scissor was used to cut off the tapered tip at the distal end of the catheter. **C**, slip the entire catheter over the syringe needle with the needle bevel exposed beyond the catheter tip, making it into a stopper for the injector. **D**, the caliper confirmed the length of the open bevel was 1 mm, a relatively safe depth for SCS injection

The SCS injector was made using commercially available, single-use materials. The injector was made using a 1-mL sterile hypodermic syringe with a 26-gauge needle (1mL, 26G × 16 mm, regular wall long bevel, Shandong Weigao Group Medical Polymer Co., Ltd, Shandong, China) and a 26-gauge intravenous catheter for single use (ZFII-B, 26G × 16 mm, Suzhou Linhua Medical, China) (Fig. 1). The intravenous catheter was sheathed onto the syringe needle. Using ophthalmic micro-scissor, the tapered tip at the distal end of the catheter was cut off, leaving 1 mm of the open bevel of the 1-mL syringe needle exposed beyond the catheter (Fig. 1A-D). The exposed bevel length of 1 mm serves as a stopper and determines the injection depth. When making the suprachoroidal injection (perpendicular to the sclera), the catheter also forms a sealed space with the sclera/conjunctiva and ensures the entire dose of the medication is delivered to the SCS without leakage. The relatively blunt tip and the controlled bevel length are intended to allow scleral penetration and drug delivery to SCS at the pars plana while reducing the risk of deeper choroidal or retinal injury.

### Surgical technique

#### Pars plana vitrectomy

The study eye received standard 25-gauge 3-port pars plana vitrectomy (PPV) using DORC EVA system (DORC, Zuidland, The Netherlands). Retrobulbar injection of 2% lidocaine (3.5mL) was administered immediately before surgery for local anesthesia. After removal of vitreous hemorrhage, intraoperative peripheral retinal photocoagulation was performed at the discretion of the surgeon (H.M). The suprachoroidal injection was performed at the end of the vitrectomy procedure.

#### Two-step suprachoroidal injection of Ozurdex

**Fig. 2:**
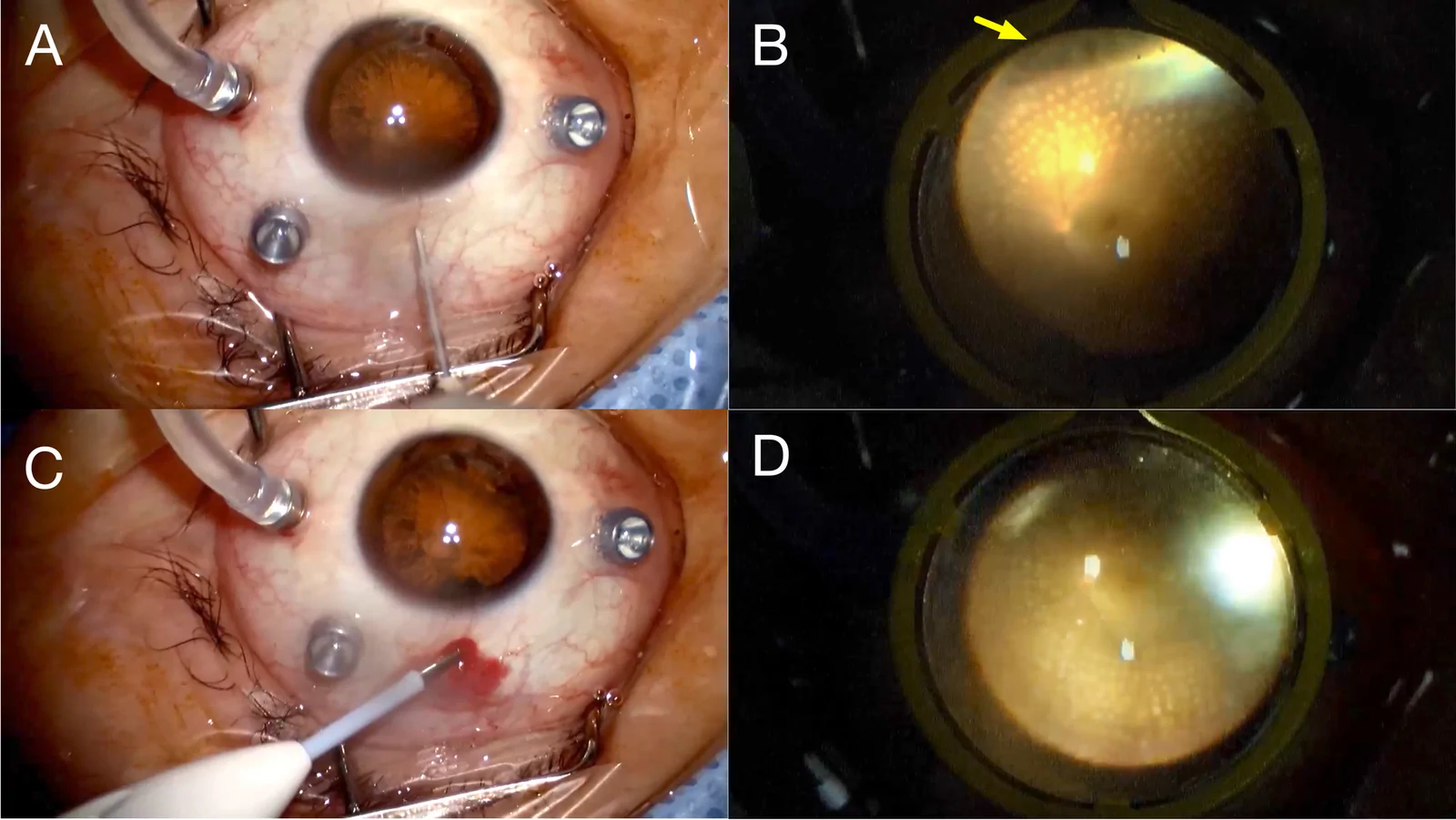
SCS injection of Ozurdex. **A**, 0.3 mL sodium hyaluronate ophthalmic viscoelastic was injected into SCS using our custom-made SCS injector 4.0 mm posterior to limbus at the 12 o’clock position. The injection was made perpendicular to the sclera with slight pressure so that the catheter and the conjunctiva could form a sealed space, ensuring that the intended amount of viscoelastic was injected into the suprachoroidal space without leakage. **B**, a focal elevation of the pars plana and peripheral choroid around the injection site (yellow arrow) was observed, indicating the presence of a safe suprachoroidal space created by the injected viscoelastic. **C**, injection of Ozurdex at the same site. A focal sub-conjunctival hemorrhage was observed. **D**, reexamination of the vitreous cavity and fundus, Ozurdex was not observed in either the vitreous cavity or the pars plana region, confirming its location within the suprachoroidal space

The suprachoroidal injection of Ozurdex consisted of two steps. Step 1: viscoelastic pre-injection. At the end of the surgery, 0.3 mL of sodium hyaluronate ophthalmic viscoelastic (20 mg/mL, Shiweike, Shanghai Qisheng Biological Preparation Co., Ltd., Shanghai, China) was injected into SCS at 12 o’clock using our custom-made SCS injector 4.0 mm posterior to limbus, a focal elevation of the pars plana and peripheral choroid was observed (Fig. 2A-B). It should be noted that the injection should be made perpendicular to the sclera with slight pressure so that the catheter and the conjunctiva could form a sealed space, ensuring that the intended amount of viscoelastic was injected into the SCS without leakage. The focal choroidal elevation was examined and assessed during surgery to ensure the space created by the viscoelastic was large enough to accommodate the 6-mm Ozurdex implant and buffer the implant injection.

Step 2: Ozurdex injection. Through the same injection site, Ozurdex was then injected to SCS using the preloaded Ozurdex injector provided by the manufacturer (Fig. 2C). Care was taken to direct the implant along the created suprachoroidal plane. After injection, the vitreous cavity and fundus was re-examined to confirm the absence of the implant in the vitreous or pars plana region, which would indicate inadvertent intravitreal placement, confirming the implant’s location within the suprachoroidal space (Fig. 2D). A surgical video demonstrating the two-step SCS Ozurdex injection technique is provided as Supplementary Material.

## Results

### Baseline clinical characteristics

**Fig. 3:**
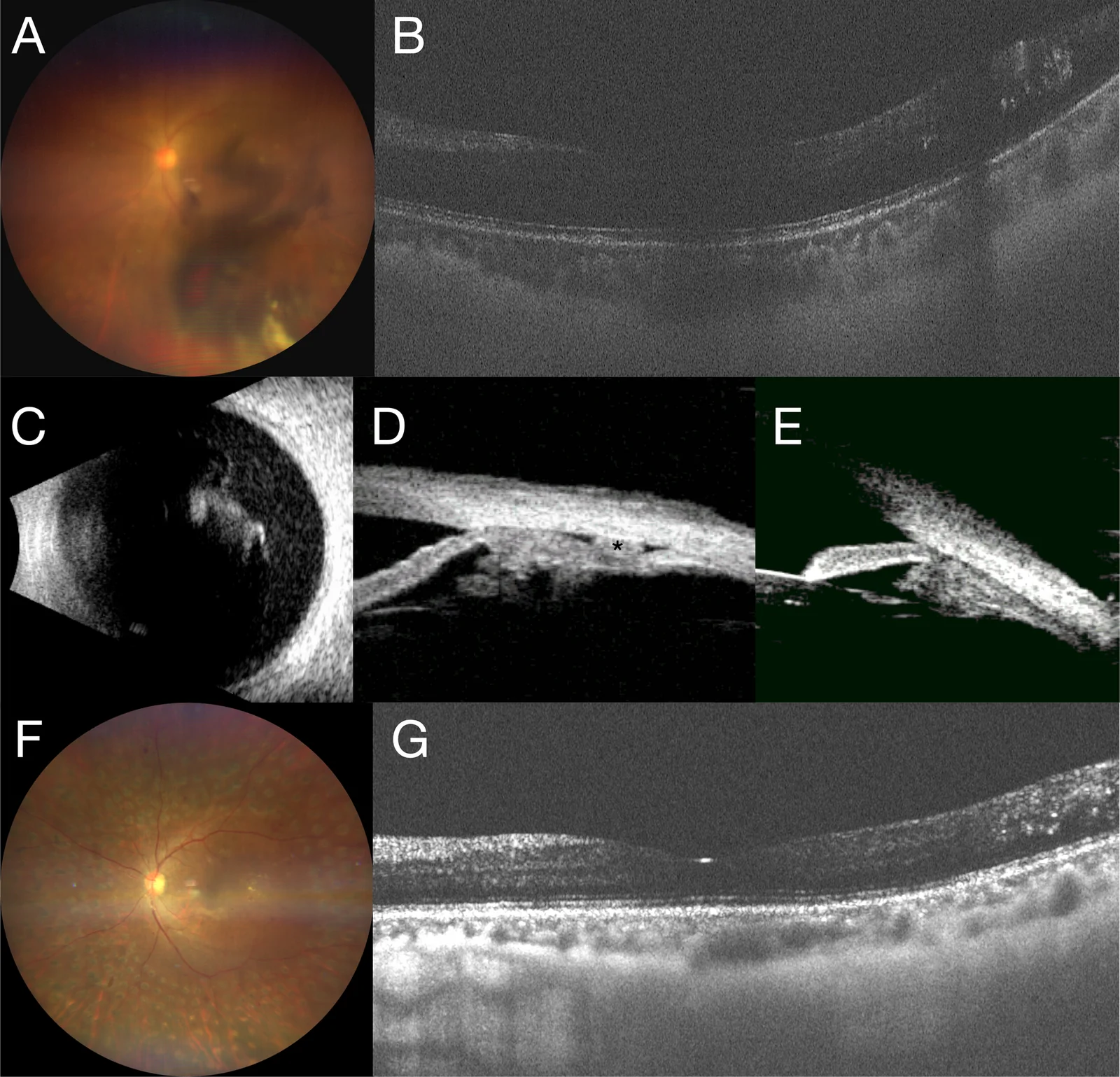
Pre- and post-operative clinical presentations of the patient’s left eye. **A**, fundus photography of the patient’s left eye, showing vitreous hemorrhage obscuring view of retina and peripheral post photocoagulation changes. **B**, OCT of left eye, revealing intraretinal cysts and hyperreflectivity. **C**, B-scan ultrasonography of left eye, revealing clumped and strand-like echoes in the vitreous cavity. **D**, UBM of left eye a week after surgery, revealing anechoic spaces between the pars plana and the sclera (12:00 position). Asterisk indicated the injected suprachoroidal Ozurdex. **E**, UBM of left eye 12 weeks after surgery, revealing the disappearance of this anechoic space, indicating the absorption of Ozurdex injected to SCS. **F**, fundus photography of left eye at 12-week follow-up, showing a clear view of retina in both eyes with few exudates and peripheral post photocoagulation changes. **G**, OCT of left eye at 12-week follow-up, revealing resolved intraretinal cyst but persistent intraretinal hyperreflectivity

A 37-year-old female at 24 weeks of gestation presented with progressive decreased vision and floaters for 6 months in her left eye. She was previously diagnosed with bilateral diabetic retinopathy with diabetic macular edema in another center and received bilaterally pan-retinal photocoagulation 3 months prior to this visit. She was diagnosed with well controlled type 2 diabetes mellitus for a year (initial fasting glucose 13mmol/L). Refraction was − 6.00 spherical diopters in both eyes. No other systemic comorbidities were reported. At baseline, BCVA in her left eye was 0.2 (Snellen 4/20), and IOP was 21 mmHg; no remarkable signs were observed in anterior segment. Funduscopy revealed vitreous hemorrhage and post-photocoagulation changes (Fig. 3A). OCT revealed macular intraretinal cysts and hyperreflectivity (Fig. 3B) though the quality of scans were compromised by vitreous hemorrhage. Based on these findings, the decision was made to perform 25G 3-port pars plana vitrectomy with intraoperative peripheral retinal photocoagulation and suprachoroidal Ozurdex injection using the two‑step technique described above.

### Feasibility and intraoperative findings

The custom‑made injector was successfully assembled from routine disposable materials. Injection of 0.3 mL sodium hyaluronate viscoelastic into the suprachoroidal space created a visible focal elevation of the pars plana and peripheral choroid at the injection site (Fig. 2B). Subsequent injection of the dexamethasone implant through the same site was accomplished without resistance. Post‑inspection of the vitreous cavity confirmed the absence of the implant in the vitreous or pars plana region, consistent with suprachoroidal placement (Fig. 2D). No intraoperative complications, including suprachoroidal hemorrhage or IOP spike, were observed.

### Short-term anatomical and functional outcomes

The patient was followed up on post-operative 1, 4 and 12 weeks. A week after PPV and SCS Ozurdex injection, UBM revealed Ozurdex in an anechoic space between the pars plana and the sclera (Fig. 3D). The 12-week post-operative UBM, however, revealed the disappearance of Ozurdex and the anechoic space (Fig. 3E). BCVA in the left eye improved from baseline 0.2 to 0.4 (Snellen 20/50) at week 12. OCT at week 12 showed resolution of intraretinal cysts, although intraretinal hyperreflectivity persisted (Fig. 3G). No signs of cataract development or progression were observed during the 12‑week follow‑up.

### Safety profile

IOP remained stable throughout follow-up: 21 mmHg (preoperative), 22 mmHg (week 1), 23 mmHg (week 4), and 21 mmHg (week 12). No clinically significant IOP elevation requiring treatment was observed in the study eye. No subjective discomfort or notable inflammatory response was reported. No delayed complications such as endophthalmitis, retinal tear or choroidal hemorrhage were observed.

## Discussion

This report describes a novel two-step technique for suprachoroidal delivery of Ozurdex using a custom-made injector assembled from routine disposable materials. The technique was successfully performed in a pregnant patient with DME following PPV, with no clinically significant IOP elevation or cataract progression during 12 weeks of follow-up. UBM confirmed suprachoroidal placement of the implant at week 1 and its disappearance by week 12, consistent with previous reports that efficacy of Ozurdex deteriorates after 3 months even if the implant could be detected as long as 6 months post-injection [6, 7].

### Comparison with existing suprachoroidal injection techniques

The only currently approved drug for SCS injection, XIPERE, requires a proprietary microinjector and is not available in many countries. Its wholesale acquisition cost of approximately $1,650 per injection [8] further limits accessibility for patients who might benefit from it. There have been reports of more cost-effective custom-made suprachoroidal injection devices for triamcinolone acetonide injection [9–11]; however, previous pharmacokinetic studies have shown that extending residence time of drugs in SCS requires their incorporation into particulate controlled release systems [12], therefore SCS injection of triamcinolone acetonide still requires repeated injections of limited intervals for persistent cases. In contrast, Ozurdex is a biodegradable solid polymer dexamethasone implant designed for controlled release of dexamethasone over several months [6, 7]. To our knowledge, there has only been one previous report of SCS injection of Ozurdex in a case of surgery-related macular edema unresponsive to intravitreal injection of triamcinolone with complete macular edema resolution and no records of ocular hypertension after two SCS Ozurdex injections (treatment interval 5 months); in their case the implant was manually inserted into the SCS through scleral incision [13]. The two‑step technique presented here differs in that it uses a custom‑made injector and viscoelastic pre‑injection, which may simplify the procedure and reduce the risk of mechanical trauma to the choroid and retina.

### Pharmacokinetic considerations

Dexamethasone is a small molecule with molecule weight of ~ 0.39 kDa that could cross the retinal pigment epithelium (RPE) through passive permeation, allowing the drug to pass through RPE into the targeted subretinal space and macula [14]. Previous studies on targeted SCS drug administration have shown strong chorioretinal selectivity of injected material, unlike intravitreal administration [1]. For dexamethasone, this could translate into reduced rates of cataract progression and steroid‑induced ocular hypertension, both of which are dose‑ and exposure‑dependent complications of intravitreal corticosteroids [15–18]. In the present case, no cataract progression or clinically significant IOP elevation was observed over 12 weeks, although longer follow‑up would be required to confirm these potential benefits.

Previous pharmacokinetic studies have shown that extending residence time of drugs in SCS requires their incorporation into particulate controlled release systems [12]. Unlike triamcinolone acetonide suspension, which is cleared more rapidly from the suprachoroidal space, Ozurdex is a biodegradable solid polymer implant composed of a poly (D, L‑lactide‑co‑glycolide) (PLG) matrix. The aqueous environment of the SCS allows gradual biodegradation of the polymer matrix, enabling comparable sustained release of dexamethasone over months (though with better chorioretinal selectivity) [19]. Although the precise release profile in the suprachoroidal space has not been directly measured, the implant remained detectable on UBM at week 1 and had disappeared by week 12 in this case, which is consistent with the known duration of dexamethasone release following intravitreal administration [6, 7]. It is worth noting that a SCS sustained-release dexamethasone acetate device (OXU-001) is being investigated and have shown promising results for the treatment of DME, indicating the bio-feasibility of our drug administration [20]. However, direct pharmacokinetic data for Ozurdex in the human SCS remain lacking, further in vitro or in vivo studies on pharmacodynamics and pharmacokinetics are required to further clarify the release profile of SCS administration of Ozurdex.

### Rationale for the two-step approach

In this report we introduced a safer and more convenient two-step delivery method with custom-made SCS injector and injection of sodium hyaluronate ophthalmic viscoelastic prior to Ozurdex injection. The use of viscoelastic pre-injection serves two purposes. First, it creates a detectable focal elevation of the pars plana and peripheral choroid, providing visual confirmation that the injector tip is positioned within the suprachoroidal space. Secondly, the viscoelastic buffer may reduce the risk of choroidal or retinal perforation during the subsequent Ozurdex injection, which is propelled with considerable force.

In our technique we opted to inject 0.3 mL of sodium hyaluronate viscoelastic prior to Ozurdex SCS injection, which is larger than the 0.1 mL typically used for SCS drug delivery (as in Xipere). However, previous studies on SCS viscoelastic injection in treatment of rhegmatogenous retinal detachment have reported up to 0.5 mL of volumn injected to SCS without significant IOP spike unresponsive to topical medications [21], and experimental models have successfully injected volumes up to 1000 µL into the anterior and posterior SCS segments without causing tissue rupture or hemorrhagic collapse as the eye could dynamically redistribute fluid circumferentially around the choroidal landscape to protect local architecture [22]. The volume of 0.3 mL was therefore considered acceptable for creating an adequate buffer space to accommodate the 6‑mm Ozurdex implant.

### Safety considerations

No intraoperative or short‑term postoperative complications were observed in this case. IOP remained within 21–23 mmHg throughout follow‑up, and no cataract progression was noted at 12 weeks. The absence of sustained IOP elevation is notable, as IOP rise is a well‑recognized complication of intravitreal corticosteroid injections. The favorable IOP profile in this case is consistent with the only other reported case of suprachoroidal Ozurdex injection [13] and may reflect reduced exposure of the trabecular meshwork via the suprachoroidal route [1].

Nevertheless, several safety concerns warrant discussion. First, the procedure in this case was performed during pars plana vitrectomy, which allows immediate management of potential complications such as suprachoroidal hemorrhage or IOP spike with the DORC Eva system. The safety and feasibility in non‑vitrectomized eyes remain unknown.

Secondly, although previous studies have reported successful suprachoroidal viscoelastic injection of 0.3–0.5 mL in non‑vitrectomized eyes, IOP elevation requiring topical medication has been observed [21]; another study reported 2 cases of central retinal artery non-perfusion out of 6 non-vitrectomized eyes after 0.6 mL SCS viscoelastic injection that were resolved after immediate anterior chamber paracentesis [23]. Therefore, the present technique is not recommended for routine use in non‑vitrectomized eyes without further evidence.

Thirdly, inadvertent intravitreal injection or choroidal perforation remains a potential risk, particularly in eyes with altered scleral anatomy. Previous clinical trials reported no serious adverse events related to suprachoroidal hemorrhage, retinal tear, or endophthalmitis for the currently commonly used suprachoroidal injection depths of 900 and 1100 μm SCS injection [4, 5, 8], suggesting 1000 μm, opted in our case, to be a relatively safe initial injection depth for viscoelastic injection. In a study on anterior ocular wall thickness by Cai et al. showed that at 4 mm posterior to the scleral spur, the anterior conjunctival-scleral thickness was 763.5 ± 82.4 μm, less than the needle length; however, this might not directly translate to procedural risk, as under physiological conditions, the stiffer structure of sclera and the more compliant choroid, along with IOP gradients, allow the needle and fluid to atraumatically create and expand the SCS without deeper penetration [24]. After viscoelastic pre-injection, the suprachoroidal space created by viscoelastic could act as a buffer and decrease the risk for choroid perforation for the upcoming Ozurdex injection. Meanwhile, pars plana, our choice for the injection site, is also a relatively avascular zone, therefore risk of mechanical trauma to pars plana during the injection process leading to choroidal hemorrhage is further mitigated. Nasal inferior anterior ocular wall thickness has also been reported to be thicker that other quadrants, therefore to reduce these possible risks, it is theoretically safer to perform the injection in the nasal inferior quadrant [24].

The potential unknown risk of toxicity of viscoelastic is also a concern. A larger case series is necessary to adequately assess the long-term safety of this procedure.

In our case, the procedure was conducted with retrobulbar block, which is our recommendation for the procedure (for both concurrent vitretomy eyes and vitrectomized eyes), as this choice of anesthesia could both reduce the pain and immobilize the eye, reducing the risk of inadvertent trauma to the choroid and retina during the procedure.

### Limitations

This study has several important limitations. First, it is a single case without a control group, which precludes definitive conclusions about efficacy or safety. Secondly, the procedure was performed immediately following PPV; therefore, the findings may not generalize to non‑vitrectomized eyes. Thirdly, direct pharmacokinetic data for Ozurdex in the human SCS remain lacking, and the potential toxicity of retained viscoelastic in the SCS has not been systematically evaluated. Larger case series and longer follow‑up are required before this technique can be recommended for routine clinical use.

### Clinical implications and future directions

Despite these limitations, the present technique may offer a practical option for selected patients with relative contraindications to intravitreal anti‑VEGF or corticosteroid therapy, such as pregnant women, patients with a natural lens who wish to minimize cataract risk or known steroid responders. The use of routinely available, low‑cost materials makes this technique accessible in resource‑limited settings where commercial suprachoroidal injectors are unavailable. Future studies should evaluate this technique in a larger cohort, with longer follow‑up and standardized IOP monitoring.

## Conclusions

We describe a two‑step surgical technique for suprachoroidal delivery of a dexamethasone implant (Ozurdex) using a custom‑made injector assembled from routine disposable materials. In this single case performed during pars plana vitrectomy, the technique appeared feasible and was not associated with short‑term complications such as sustained intraocular pressure elevation, cataract progression, or implant misplacement. This technique may offer a low‑cost, readily accessible alternative for selected patients with non‑infectious macular edema who have relative contraindications to intravitreal anti‑VEGF or corticosteroid therapy. However, the present findings are preliminary; larger case series and longer follow‑up are required before routine clinical application can be recommended.

## Supplementary Information

Below is the link to the electronic supplementary material.


Supplementary Material 1


## Data Availability

No datasets were generated or analysed during the current study.

## Abbreviations

BCVA: Best corrected visual acuity
DME: Diabetic macular edema
IOP: Intraocular pressure
OCT: Optical coherence tomography
PDR: Proliferative diabetic retinopathy
PPV: Pars plana vitrectomy
SCS: Suprachoroidal space
UBM: Ultrasound biomicroscopy
VEGF: Vascular endothelial growth factor
